# Serological and molecular survey of *Leptospira* spp. infections in wild boars and red foxes from Southeastern France

**DOI:** 10.14202/vetworld.2021.825-828

**Published:** 2021-04-02

**Authors:** Cédric Roquelo, Angeli Kodjo, Jean-Lou Marié, Bernard Davoust

**Affiliations:** 1 French Military Health Service, Saint Denis, La Réunion, France; 2 Animal Epidemiology Expert Group, French Military Health Service, Tours, France; 3 Leptospirosis Laboratory, Veterinary Campus of Lyon, VetAgro Sup, Marcy l’Etoile, France; 4 Expertise and Defense Health Strategy Division, French Military Health Service, Paris, France; 5 Aix Marseille Univ, IRD, AP-HM, MEPHI, Marseille, France; 6 IHU Méditerranée Infection, Marseille, France

**Keywords:** France, *Leptospira* spp, red fox, wild boar

## Abstract

**Background and Aim:**

Leptospirosis is a zoonotic disease. Information on the recent prevalence of *Leptospira* in hunted wild animals is limited, particularly in southeastern France. A cross-sectional survey was conducted to assess the prevalence and diversity of *Leptospira* spp. among wild boars (*Sus scrofa*) and red foxes (*Vulpes vulpes*) from two military camps in Southeastern France.

**Materials and Methods:**

Serological analyses were performed using microscopic agglutination tests and polymerase chain reaction (PCR) assays were used to demonstrate *Leptospira* spp. infection from boar kidney DNA extracts.

**Results:**

According to the species, the positive sera were obtained from 18% of 358 boars and 6 % of 64 foxes tested. The prevalence rate is significantly higher (p≤0.02) in boars than in foxes. In wild boar, Australis represents the most recorded serogroup (15.9%), followed by Sejroe (2.8%) and icterohaemorhagiae (2.8%). In red fox, icterohaemorhagiae represents the most recorded serogroup (6.25%), followed by Sejroe (1.57%) and Hebdomadis (1.57%). PCR-based detection of *Leptospira* DNA was positive in 6/62 (9.6%) of the wild boars tested.

**Conclusion:**

The results of this study confirmed the importance of wild boar in the epidemiology of leptospirosis among wildlife in Southeastern France. Due to their predatory behavior and their varied diet, mainly composed of small mammals, red foxes could be considered sentinel animals of environmental contamination with leptospires.

## Introduction

Leptospirosis is a worldwide zoonotic disease caused by spirochetes of the genus *Leptospira*. The genus *Leptospira* comprises 35 species that are divided into more than 300 serovars [1]. Among these species, ten are pathogenic and can infect and cause disease in humans and animals (*Leptospira interrogans*, *Leptospira borgpetersenii, Leptospira santarosai, Leptospira noguchii, Leptospira weillii, Leptospira kirschneri, Leptospira alexanderi*, *Leptospira mayottensis*, *Leptospira alstonii*, and *Leptospira kmetyi*), and five are known as intermediates and may cause various mild clinical manifestations of leptospirosis [2]. Leptospirosis is an important but neglected disease. A study based on a systematic review of published morbidity and mortality studies and databases estimated, using modeling, that there were annually 1.03 million cases (95% CI 434,000-1,750,000) and 58,900 deaths (95% CI 23,800-95,900) due to leptospirosis worldwide [3]. Autochthonous leptospirosis is an emerging zoonotic disease in Europe, particularly in France. In France, the incidence of all forms of leptospirosis is one of the highest in Europe with an increase in the number of cases since 2014, that is, 600 cases per year with an incidence of one case per 100,000 inhabitants [4]. Humans are infected by contact with infected animal tissues or urine, contaminated water, or soil. Many wildlife species have been implicated as reservoirs for the bacteria, including roe deer (*Capreolus capreolus*), red fox (*Vulpes vulpes*), and wild boar (*Sus scrofa*) [5].

Swine, including wild boar and pig, is recognized as maintenance hosts for Pomona, Tarassovi, and Bratislava serovars [6], and fox is recognized as maintenance host for icterohaemorhagiae and Ballum. Due to their high adaptability, wild boar populations have increased over the last decades in Europe. In France, where the evolution of wild boars’ population is monitored through the examination of bags, a constant and significant increase has been observed for more than 20 years, especially in the south [7]. The red fox is the most widespread and abundant wild carnivores in Europe. It is widely distributed in the northern hemisphere, including metropolitan France.

Information on the recent prevalence of *Leptospira* in hunted wild animals is limited, particularly in southeastern France, and almost non-existent for the military camp. The aim of the present study was to investigate the prevalence of *Leptospira* with serological and molecular assays among wild boars and foxes in Southeastern France.

## Materials and Methods

### Ethical approval

The samples were taken from dead animals that had just been shot by hunters. So, ethical approval was not necessary.

### Study area and period

This study was carried out in the Departments of Bouches-du-Rhône and Var in Southeastern France, on military camp of Canjuers (43° 38’ 49” North; 6° 27’ 56” East) and on military camp of Carpiagne (43° 14’ 54” North; 5° 30’ 43” East). Wild boar, roe deer, and red fox are the main wild large mammalian species in the area. Samples were collected during 2015 and 2016 hunting season (from 15 September to 31 January).

### Animals and sampling

A military hunting society is authorized to regulate the population size of wild boar, roe deer, and red fox. Blood samples collected on blotters were obtained from 64 hunted red foxes (49 from Carpiagne and 15 from Canjuers) and 358 wild boars (23 from Carpiagne and 335 from Canjuers). Kidney samples from wild boars were collected and were kept at −20°C until DNA extraction.

### Serological analysis

Serological analyses were performed at the French Leptospira laboratory in Lyon using microscopic agglutination tests (MAT) with a dilution threshold of 1:20 of 25 leptospire serovars for red foxes. Due to the dual approach (serology and polymerase chain reaction [PCR]) and the low sensitivity of the MAT test using blotters, we chose to modify the MAT diagnostic approach for wild boars by limiting the dilution of the blotter to 1:10 and by limiting the serogroups tested to those potentially present in France (Icterohaemorhagiae, Australis, Autumnalis, Ballum, Canicola, Panama, Pyrogenes, Sejroe, Cynopteri, and Grippotyphosa). Positive responses correspond to sera for which contact with leptospires can be epidemiologically objectified.

### Molecular biology

PCR assays were used to demonstrate *Leptospira* spp. infection from DNA extracts of boar’s kidney [8,9]. PCR was only performed in MAT positive wild boars. Sixty-six DNA samples were delivered to the French Leptospira laboratory in Lyon (France). The analysis was carried out in three steps: Identification of positive DNAs by PCR on 16S rDNA, typing of Leptospira species on positive DNAs, attempt to identify the serovar involved by Variable Number Tandem (VNTR). Briefly, after extraction of DNA on Qiagen column (Qiagen, Hilden, Germany), end-point PCR reactions were carried out on pure and 10-fold dilution of the DNA according to the standardized protocol of the laboratory. The presence of an amplification between 300 and 400 base pairs on 1.5% agarose gel after migration was considered a positive signal. This method has been validly used in previous study to explore the renal carrier state in a very large variety of wildlife mammals [10].

### Statistical analysis

The prevalence was calculated as the number of animals with positive serum devised by the total number of animals studied in each group. The prevalence rates were compared between species and location using Chi-square when the number of observations was sufficient, or the Fisher exact test when it was not. Odds ratios (OR), 95% confidence interval (CI) and p values were calculated separately for each variable. p ≤ 0.05 was considered significant. All statistical tests were carried out using the Epi Info Software (7.1.3.0 version, CDC Atlanta, USA).

## Results

Of a total number of 422 animal blood samples, 16.6 % (CI 95: 13.0-20.1) tested positive with one or several serovars of pathogenic *Leptospira*. Table-1 illustrates the prevalence according to the respective species. According to the species, the positive sera were obtained from 18% of 358 boars and 6% of 64 foxes tested. The prevalence rate is significantly higher (p≤0.02; OR: 3.39 [1.19-9.66]) in boars than in foxes. According to species, no correlation was found between seroprevalence and location (p>0.5). Positivity can be observed for one or more leptospiral antigens, so we have recorded 88 positive serological reactions with different serovars for the 70 positive samples. This phenomenon is due, in general, to the presence of co-agglutinins in sera, but does not exclude the possibility that the animal is infected simultaneously with several strains or keeps a serological trace of prior(s) infection(s). In wild boar, Australis represented the most recorded serogroup (15.9%), followed by Sejroe (2.8%) and icterohaemorhagiae (2.8%). In red fox, icterohaemorhagiae represented the most recorded serogroup (6.25%), followed by Sejroe (1.57%) and Hebdomadis (1.57%). Of the positive serological reactions, the most frequently serogroups recorded were, respectively, Australis (69.5%) in wild boar and icterohaemorhagiae (66.7%) in fox.

**Table-1:** Percentage of positive samples for leptospirosis according to species.

Animals	Samples	Test	Canjuers		Carpiagne		Total	
		
			Number	Positives	Number	Positives	Number	Positives
Boars	Blood	MAT	335	59 (17.6%)	23	7 (30.4%)	358	66 (18.4%)
	Kidney	PCR 16S	55	6 (10.9%)	7	0	62	6 (9.6%)
Foxes	Blood	MAT	15	0	49	4 (8.2%)	64	4 (6.2%)
Total	Blood	MAT	350	59 (16.8%)	72	11 (15.2%)	422	70 (16.5%)

MAT=Microscopic agglutination tests, PCR=Polymerase chain reaction

PCR-based detection of *Leptospira* DNA was positive in 6/62 (9.6%) of the wild boars tested. Only the kidneys of the 62 seropositive boars were sent to the laboratory for PCR analysis. Initially, we wanted to perform PCR on all kidney samples, but for cost and time reasons PCR was only performed on seropositive wild boar. We are aware that there is a bias in our study due to this lack of resources. Sequences of amplified DNA fragment by the 16S rDNA PCR allowed species identification after alignment with all 16S rDNA sequences of all known *Leptospira* species and result allows the identification of *L. interrogans*. Despite multiple attempts, serovar identification by VNTR analysis failed.

## Discussion

Among wildlife, wild boar is an important *Leptospira* reservoir and could represent an appropriate indicator for this zoonotic infectious disease. The military camps offer particularly favorable conditions for the proliferation of wild boars (tranquility and large space). Among the samples collected, 18.4% of the 358 wild boars hunted are seropositive toward one or several serovars of pathogenic leptospires. However, the cutoff points selected (low in epidemiological investigation), and the absence of kinetic serology does not allow in most cases to conclude to a current active infection. Although differences in method (threshold, untested serogroups,…) limit the relevance of comparison with other studies, the seroprevalence reported in this investigation was very close to other data obtained in recent studies performed in different areas of Europe (13.6% in Central Italy; and 10.4% in 16 polish provinces) [11,12]. According to the literature, the distribution of serovars in wild boar in Europe is not homogeneous. In our study, the Australis serogroup is the most represented as in central Italy [11].

Very few studies were carried out on pathogenic Leptospira DNA in wild boar kidneys. The kidney is an immunoprivileged environment and leptospires that replicate in this organ are not exposed to the same constraints that are imposed by the host immune response in other tissues. In our study, pathogenic *Leptospira* DNA was detected in six of 62 seropositive wild boar kidneys. Pathogenic *Leptospira* DNA in wild boar kidneys belongs to *L. interrogans*. The identification of the serovar based on molecular strategy failed due to the fact that the amount of Leptospira DNA in the extracted DNA was low, and this has already been pointed out by the previous studies [13]. Isolation of leptospires from kidneys suggests that positive wild boars are chronic renal carriers and could contaminate their environment. This renal portage is probably underestimated in our study because only the kidneys of seropositive wild boar have been tested and seronegativity of *Leptospira*-positive kidneys has already been reported for other animal species, including pigs, suggesting early, or chronic infection [14,15].

Considering wild boar behavior and its ability to live in a wide spectrum of habitat types including sub-urban areas, a transmission between human and wildlife could be possible. Hunters from military hunting companies are particularly exposed to a high risk of infection. They handle, sometimes without precautions, the carcasses and their families usually consume the hunted animals directly.

Among the 64 hunted red foxes, only 4 (6%) were serologically positive toward one or several serovars of pathogenic leptospires, mainly for the serovar icterohaemorhagiae. Red foxes’ prey on small rodents, notably *Rattus norvegicu*s, which is known to be the main reservoir of icterohaemorhagiae serovar. Surveys conducted throughout Europe have shown differences in the prevalence of leptospirosis in foxes: 26.3% in central and eastern Poland [16], 31.3% in Croatia [17]. The low percentage of seropositive foxes in our investigation is probably due to the use of MAT on blotting papers, which is a less sensitive method, but also probably reflects the biotic structure of leptospirosis foci in Southeastern France and the myomorphous mammals’ population. The leptospiral infection in foxes would depend on the spread of leptospires among small mammals.

Although in our study wild boars and red foxes were hunted during the same period in the same geographical area (Carpiagne and Canjuers), there is no significant correlation between the main serogroups found in red foxes (Icterohaemorhagiae) and wild boars (Australis). This could be explained by different exposure to different serovars related to their living habits (diet, behavior…) and a different sensitivity to different serovars.

## Conclusion

The results of this study confirmed the importance of wild boar in the epidemiology of leptospirosis among wildlife in southeastern France. The relatively high prevalence of *Leptospira* spp. infections in wild boars may constitute a threat, in particular to hunters and forestry workers. Due to their predatory behavior and their varied diet, mainly composed of small mammals, red foxes could be considered sentinel animals of environmental contamination with leptospires.

## Authors’ Contributions

CR prepared the manuscript. JLM and BD designed the study and carried out the sample collection. AK carried out laboratory analyses. AK, JLM, and BD carried out proofreading and made critical comments on the manuscript. All authors read and approved the final version of the manuscript.
